# Evaluation of the Interaction of Cinacalcet with Calf Thymus dsDNA: Use of Electrochemical, Spectrofluorimetric, and Molecular Docking Methods

**DOI:** 10.3390/bios12050278

**Published:** 2022-04-27

**Authors:** Cem Erkmen, Didem Nur Unal, Sevinc Kurbanoglu, Gokcen Eren, Bengi Uslu

**Affiliations:** 1 Department of Analytical Chemistry, Faculty of Pharmacy, Ankara University, Ankara 06560, Turkey; cerkmen@ankara.edu.tr (C.E.); dnunal@ankara.edu.tr (D.N.U.); skurbanoglu@ankara.edu.tr (S.K.); 2 The Graduate School of Health Sciences, Ankara University, Ankara 06110, Turkey; 3 Department of Pharmaceutical Chemistry, Faculty of Pharmacy, Gazi University, Etiler, Ankara 06330, Turkey; gokcene@gazi.edu.tr

**Keywords:** calf thymus double-stranded deoxyribonucleic acid, cinacalcet, electrochemistry, fluorescence spectroscopy, molecular docking

## Abstract

The binding of drugs to DNA plays a critical role in new drug discovery and is important for designing better drugs. In this study, the interaction and binding mode of calf-thymus double-stranded deoxyribonucleic acid (ct-dsDNA) with cinacalcet (CIN) from the calcimimetic drug that mimics the action of calcium on tissues group were investigated. The interaction of CIN with ct-dsDNA was observed by the differential pulse voltammetry (DPV) technique by following the decrease in electrochemical oxidation signals to deoxyguanosine and adenosine. A competitive study was performed on an indicator, methylene blue, to investigate the interaction of the drug with ct-dsDNA by fluorescence spectroscopy. Interaction studies have shown that the binding mode for the interaction of CIN with ct-dsDNA could be groove-binding. According to the results obtained, the binding constant values were found to be 6.30 × 10^4^ M^−1^ and 3.16 × 10^5^ M^−1^, respectively, at 25 °C as obtained from the cyclic voltammetry (CV) and spectroscopic techniques. Possible molecular interactions of CIN with dsDNA were explored via molecular docking experiments. The docked structure indicated that CIN could fit well into the minor groove of the DNA through H-bonding and π-π stacking contact with CIN.

## 1. Introduction

Secondary hyperparathyroidism (SHPT) is a disease caused by an increase in parathyroid hormone (PTH), excessive parathyroid gland hyperplasia, and abnormality in calcium and phosphorus balances in bone mineral metabolism. In addition, SHPT often develops in chronic kidney disease patients requiring hemodialysis [1,2]. Calcium and phosphate released from bones increases mortality in patients. Therefore, it is critical to control serum PTH, minerals, and bone metabolic marker levels in SHPT patients undergoing hemodialysis [2,3]. In addition, although it is rare, parathyroid carcinoma, which is a devastating malignancy, is explained by elevated serum calcium and PTH levels. Calcium-lowering agents are needed in patients with difficult-to-treat parathyroid carcinoma [4].

**Scheme 1:**
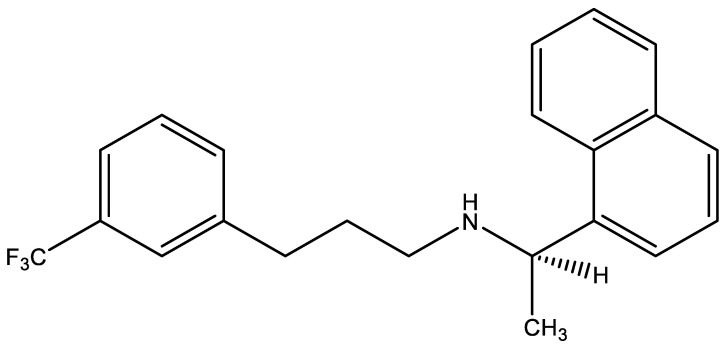
Molecular structure of CIN.

Cinacalcet (CIN, Scheme 1) belongs to the calcimimetic drug group. It activates calcium-sensing receptors of extracellular calcium in the parathyroid glands. The use of CIN reduces PTH production and regulates the calcium and phosphorus balance in the serum [3,5]. Ari et al. investigated the effect of CIN on oxidative stress related to deoxyribonucleic acid (DNA) damage in maintenance hemodialysis patients with SHPT [6]. As a result of the study, reduction in oxidative stress, improvement in antioxidant protection, and endothelial function were observed after 6 months of CIN treatment in hemodialysis patients. The overall decrease in oxidative stress with CIN treatment may be due to improved mineral metabolism or unknown pleiotropic effects of the drug [6]. Moe et al. tested that single-nucleotide polymorphisms in the calcium-sensing receptors altered the response to CIN with a patient’s DNA sequences. According to the obtained results, they reported that the differences in the biochemical reaction to CIN in many patients can be partially explained [7].

DNA is a basic biological structure responsible for various biological activities of living things. Studying the interaction of DNA with various biochemical molecules and drugs is very important and plays a critical role in new drug discoveries [8,9,10,11]. In the studies of Palecek et al., the electrochemical properties of DNA nucleic acids were elucidated and became the basis for the development of DNA sensors [8,9]. With the electrochemical oxidation of nucleic acids belonging to DNA, DNA biosensors are being developed for different application areas [10,11,12,13,14]. Electrochemical examination of the interactions of drugs with DNA for new drug discoveries is another area that benefits from the electrochemical properties of DNA [15,16,17,18,19]. When the interactions between the drug and DNA are analyzed using analytical techniques such as electrochemical and spectroscopy, important information is obtained about the drug’s binding mode to DNA. These interaction types are mainly studied in four groups: (i) direct covalent binding of the drug with DNA; (ii) electrostatic interaction, electrostatic attraction with the anionic sugar-phosphate backbone of DNA; (iii) groove binding, interactions with the DNA groove; and (iv) intercalation between base pairs [20,21,22].

In the literature, several studies were reported for CIN analysis using reversed-phase liquid chromatography [23], reversed-phase high-performance liquid chromatography [24], and spectroscopic methods [25]. Given the biological importance of CIN, it is important to obtain information about its interaction with DNA. No previous study has been reported in the literature on the investigation of the interaction of CIN with DNA using electrochemical and spectroscopic techniques. In this scope of work, the interaction between CIN and ct-dsDNA was examined electrochemically by DPV technique with, as the most common carbon electrode, the glassy carbon electrode (GCE). The interaction was investigated both after creating a biosensor on the GCE surface and in the acetate buffer solution (ABS, pH 4.7). In addition, the interaction of CIN with polyadenine (polyA) was investigated by DPV. Furthermore, the interaction between CIN and ct-dsDNA was explored using the fluorescence spectroscopic technique, and the binding constant was calculated by analyzing the thermodynamic data of the interaction. The results, which were well supported by the molecular docking analysis, showed strong binding between CIN and DNA, and important information about the binding mode was also obtained.

## 2. Material and Methods

### 2.1. Materials

CIN was provided from NOBEL İLAÇ SAN. VE TİC. A.Ş. (Düzce, Turkey). Methylene blue (MB), sodium acetate, acetic acid, sodium hydroxide, ct-dsDNA, and polyA were purchased from Sigma Aldrich (St.Louis, MA, USA). Stock solutions of the supporting electrolyte were made using analytical grade reagents and ultra-pure water from a Millipore Milli-Q system. Spectrofluorometric and electrochemical measurements were performed using 0.1 M ABS, pH 4.7, as a supporting electrolyte. All the solutions prepared were stored at 4 °C.

### 2.2. Preparation of Solutions

The stock solution of CIN was prepared in ultra-pure water and the required dilutions were prepared using ABS in all studies. The ultra-pure water was used to prepare both stock solutions and working solutions of the desired concentration of ct-dsDNA, polyA, and MB. Sodium acetate and acetic acid solutions of 0.1 M each were mixed to prepare ABS. Ultraviolet-visible (UV-vis) absorption measurements were performed to calculate the stock solution’s concentration of the ct-dsDNA using the Lambert—Beer’s Law equation (A = εbc) at 260 nm. In this equation, A, ε, b, and c define the absorption value, the molar absorption coefficient, the cuvette path length, and the concentration of ct-dsDNA, respectively.

### 2.3. Apparatus

The conventional three-electrode system was used to obtain electrochemical signals for all electrochemical studies. The GCE (*φ* = 3.0 mm) was used as a working electrode, the reference electrode consists of Ag/AgCl (BASi; 3 M NaCl), and an auxiliary electrode was a platinum wire. AUTOLAB-PGSTAT 204 (Eco Chemie, Utrecht, The Netherlands) running with NOVA 2.1.4 software was used to perform all voltammetric measurements. A fluorescence spectrophotometer (Agilent Cary Eclipse Fluorescence Spectrophotometer) was used for fluorescence measurements and the sample was placed in a 10 mm × 10 mm dimensions quartz cuvette. UV-vis absorption spectrophotometer (Shimadzu 1601PC double beam) was used for the determination of the concentration of ct-dsDNA. Atomic force microscope (AFM) analysis was carried out by the ezAFM (NanoMagnetic Instruments, Oxford, UK) model with ezAFM v.6.15 software in the tapping mode. Probe series ACLA model cantilever from AppNano, nominal frequency 190 kHz and force constant 58 N m^−1^ were used. The vibration amplitude 2.0 V_RMS_ and free vibration amplitude 1.7 V_RMS_ were applied. In this analysis, AFM images with a resolution of 256 × 256 were obtained.

### 2.4. Methods

#### 2.4.1. Electrochemical Measurements

The electroanalytical studies were carried out by DPV using anodic scan mode and the following experimental conditions were set as the potential scan range from 0.0 V to +1.60 V, pulse amplitude of 50 mV, step potential of 5 mV, and scan rate of 50 mV s^−1^. Before commencement of each measurement, the surface of GCE was thoroughly polished with alumina slurry (particle size: 0.05 μm) on a polishing pad. The biosensor was prepared to form 5 multilayers by dropping 5 µL of 50 ppm ct-dsDNA on the surface of GCE and allowed to dry. Before using the biosensors, the unbounded ct-dsDNA molecules were removed by rinsing the GCE surface with double distilled water.

#### 2.4.2. Fluorescence Measurements

The fluorescence spectra were recorded with different concentrations of CIN in the complex formed by adding 50 μM MB and 100 μM ct-dsDNA. Experimental parameters were as follows: excitation wavelength = 670 nm, excitation and emission slits = 5 nm, photomultiplier tube voltage = 600 V. The fluorescence experiments were performed at three different temperatures (288 K, 298 K, and 308 K), and the sample temperature was regulated by a Peltier temperature controller.

#### 2.4.3. Molecular Docking

Molecular docking studies were performed using the Glide docking protocol implemented in the Schrödinger Small-Molecule Drug Discovery Suite (Small-Molecule Drug Discovery Suite 2020-4, Schrödinger, LLC, New York, NY, USA, 2020). The builder panel in Maestro was used to obtain the compound structure. Then, under default conditions, ligand preparation was carried out using LigPrep (Schrödinger Release 2020-4: LigPrep, Schrödinger, LLC, New York, NY, USA, 2020). The X-ray crystal structure of B-DNA dodecamer d(CGCGAATTCGCG)2 (PDB:1BNA) was downloaded from the Protein Data Bank [26] and its protein structure was prepared by the Protein Preparation Wizard tool. Firstly, hydrogen atoms were added to the structure, then all-atom charges and atom types were by assignment. In the final step, the OPLS3e force field was applied for energy minimization and refinement of the structures up to 0.3 Å RMSD [27]. The centroid of the residues, predicted by SiteMap, was defined as the grid box and vdW radius scaling factor 1.00, partial charge cutoff 0.25, and OPLS3e force field were used for receptor grid generation. The compound prepared by LigPrep was docked into the DNA using the extra-precision (XP) docking mode of the Glide without using any constraints. A 0.85 vdW radius scaling factor,0.20 partial charge cutoff, and the post-docking minimization was employed [28]. The lowest XP glide score was used to determine the best conformation.

#### 2.4.4. Characterization of ct-dsDNA/GCE with AFM

AFM images were used to analyze the structural properties of the multilayer film ct-dsDNA modified to the glass surface. To assess the thickness of the biofilm, the clean glass surface was coated with 5 µL of 5 drops each containing 50 ppm of ct-dsDNA and investigated by AFM. The thickness of the biofilm covering with ct-dsDNA was then measured.

## 3. Results and Discussion

### 3.1. Electrochemical Interaction of CIN and ct-dsDNA/GCE

The principle of preparing electrochemical DNA biosensors is based on the immobilization of DNA on the surface of the working electrodes [13,18,29,30]. The DNA-based biosensor prepared by immobilizing the DNA on the electrode surface interacts with the analytes in the solution, enabling the elucidation of the mechanism of action of the drug molecules by drug–DNA interactions [11,31,32]. During the interaction, necessary interpretations can be concluded by monitoring changes in the electrochemical signals of electroactive deoxyguanosine (dGuo) and deoxyadenosine (dAdo) bases in the structure of DNA or the drug molecules. Among voltammetric methods, DPV allows the evaluation of more sensitive, reproducible, and accurate results [33]. As can be seen in the next section, CIN exhibited the irreversible oxidation mechanism in ABS (pH 4.7) using the conventional CV method. In this study, the CV peak is characterized by the peak potential value of about 1.018 V, and this peak can be attributed to the CIN oxidation process.

**Figure 1:**
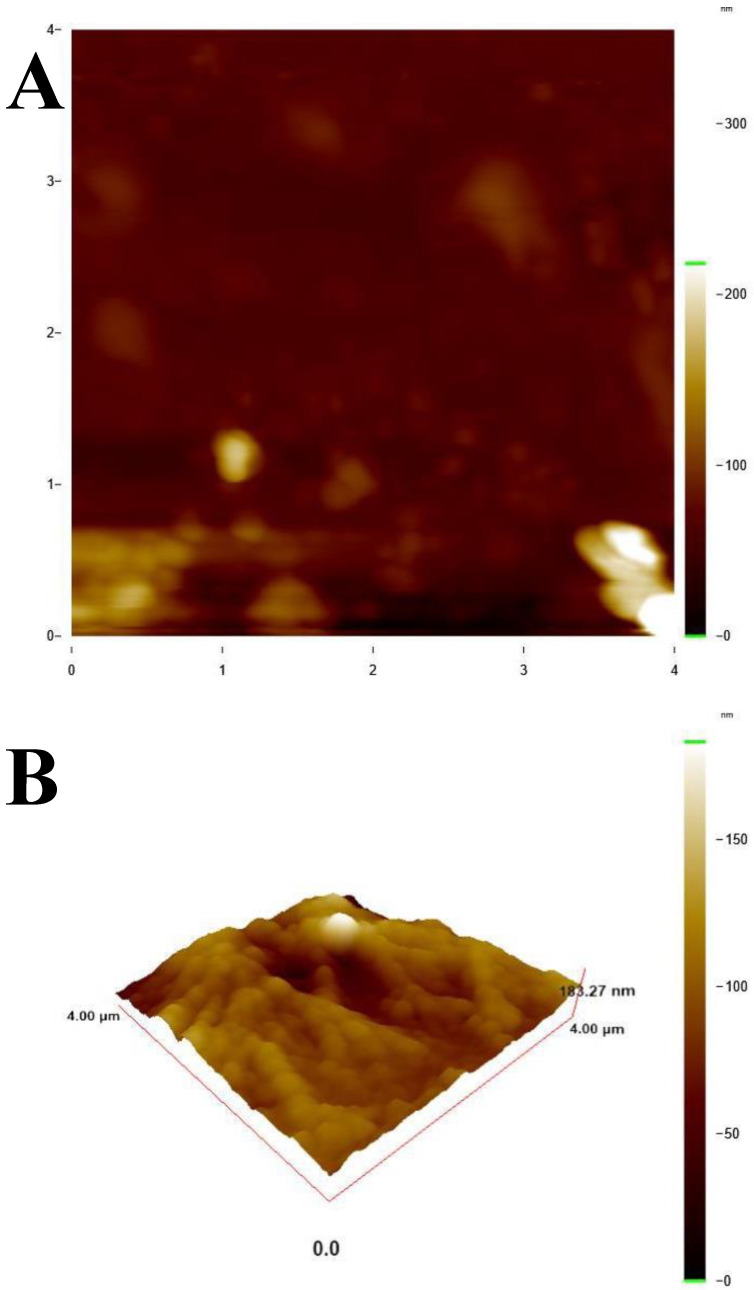
AFM topographical images (**A**) Clean glass surface, (**B**) Multilayer ct-dsDNA 3D view 4 × 4 μm scan size.

Before starting the interaction studies, the reproducibility of five different ct-dsDNA biosensors prepared on the same day and on different days were evaluated. Considering the DPV responses for dGuo and dAdo, intraday and interday relative standard deviation (RSD%) values were found to be 2.4% and 2.8% for dGuo, and 2.2% and 2.6% for dAdo, respectively. These results confirmed that the prepared disposable ct-dsDNA biosensor can be used efficiently for CIN interaction. Moreover, the ct-dsDNA biofilm thickness on the glass surface was investigated by AFM. From Figure 1A, a clean surface without ct-dsDNA film was shown. When the glass surface was coated with five layers of 5 µL of 50 ppm ct-dsDNA, the biofilm thickness increased to 183.27 nm as seen in Figure 1B. Comparing Figure 1A,B, it was confirmed that the surface was covered with ct-dsDNA. Thus, it was seen that the electrode (Section 2.4.1) prepared with 50 ppm concentration five multilayer ct-dsDNA forms a suitable film for interaction studies.

**Figure 2:**
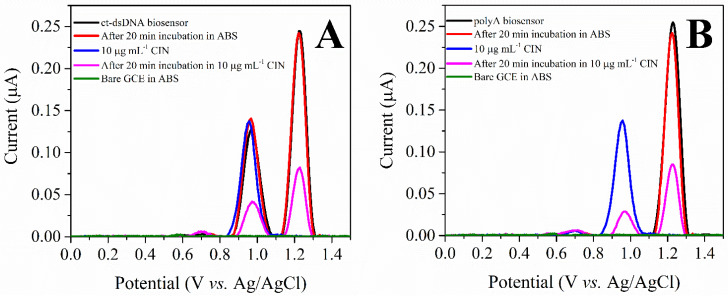
(**A**) DP voltammograms of the ct-dsDNA biosensor, 10 μg mL^−1^ CIN, the ct-dsDNA biosensor after incubation in ABS (pH 4.7), the ct-dsDNA biosensor after incubation in 10 μg mL^−1^ CIN, and the bare GCE in ABS (pH 4.7), (**B**) DP voltammograms of polyA biosensor, 10 μg mL^−1^ CIN, the polyA biosensor after incubation in ABS (pH 4.7), the polyA biosensor after incubation in 10 μg mL^−1^ CIN, and the bare GCE in ABS (pH 4.7).

As shown in Figure 2A, the anodic behavior of the prepared multilayer ct-dsDNA biosensor exhibited two well-defined anodic peaks. While an oxidation peak at 1.010 V was monitored for dGuo, the oxidation peak of dAdo was observed at 1.229 V. The DP voltammogram of 10 μg mL^−1^ CIN at the bare GCE exhibited (curve blue line) one oxidation peak at 0.992 V. As can be concluded from these results, the oxidation peaks of CIN and dGuo overlapped, therefore the interaction between CIN and ct-dsDNA was investigated through the changes in the dAdo signal. The interaction of CIN and ct-dsDNA was detected after 20 min incubation of the ct-dsDNA biosensor in 10 μg mL^−1^ CIN (curve pink line), where a clear decrease in the peak current was observed. In addition, the prepared ct-dsDNA biosensor was kept in pH 4.7 ABS for 20 min to ensure the interaction was between CIN and ct-dsDNA and not from ABS. The absence of any change in the dAdo signal (curve red line) confirmed the interaction of CIN with ct-dsDNA.

To confirm a valid interaction mechanism, 5 µL of 50 ppm polyA (five multilayers) was immobilized onto the GCE surface as in the ct-dsDNA biosensor and its interaction with CIN was investigated. As seen in Figure 2B, the anodic response of dAdo decreased upon interaction with CIN. The signal reduction obtained with the same CIN concentration and the same incubation time was similar to the amount of decrease observed in the aforementioned ct-dsDNA biosensor, thus confirming the electrochemical interaction through dAdo with CIN.

The DPV anodic oxidation peak current of the dAdo was expressed as the relative value (S%) using the following Equation (1) and compared to that obtained at the ct-dsDNA biosensor before the interaction with CIN.

(1) $$S\%=\frac{S_{1}}{S_{0}}\times 100$$

where, *S*_0_ is the current signal of dAdo recorded at the ct-dsDNA biosensor, and *S*_1_ is the current signal of dAdo after interaction with CIN.

**Figure 3:**
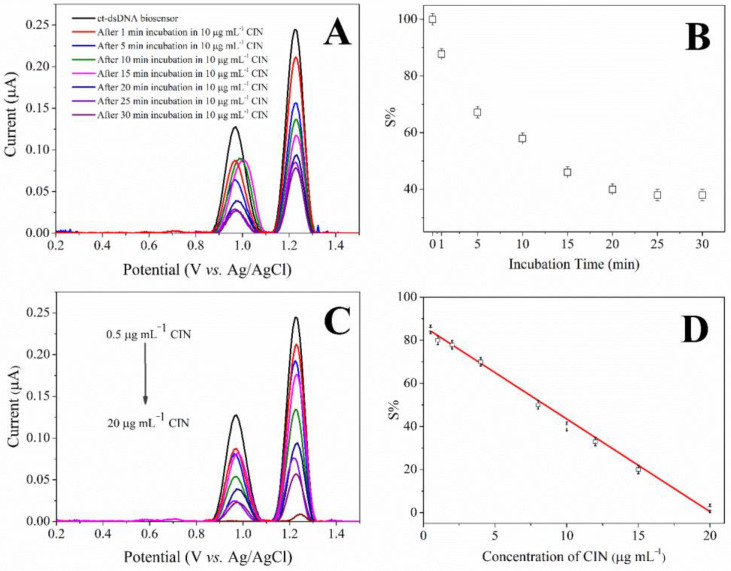
(**A**) DP voltammograms and (**B**) the plot of the current of the dAdo peak vs. incubation time for the interaction of ct-dsDNA biosensor with 10 μg mL^−1^ CIN, (**C**) DP voltammograms of the ct-dsDNA biosensor after interacting with various CIN concentrations, (**D**) Calibration plot for CIN determination based on the current of the dAdo peak.

Firstly, the effect of interaction time on the ct-dsDNA biosensor for CIN was investigated. The reduction in dAdo peak signal over 30 min was recorded (Figure 3A). According to the relative obtained values, a decrease was observed in the relative values with increasing incubation time (Figure 3C). The optimum interaction time was found to be 20 min, at which the interaction remained constant under the given conditions. Figure 2B showed that the anodic current of the dAdo moiety of ct-dsDNA decreased with increasing CIN concentration after 20 min of interaction. These results can be attributed to the strong binding of CIN to the electroactive DNA base. Moreover, the results obtained demonstrated the potential to develop an electroanalytical method for the determination of CIN using the DNA biosensor. In the prepared ct-dsDNA biosensor, at 1.229 V, the DPV current of dAdo decreased linearly with increasing CIN concentration between 0.5 μg mL^−1^ and 20 μg mL^−1^. In addition, the relationship between the S% of the oxidation peaks for dAdo and the CIN concentration can be described well with the following linear equation: *S*% = −4.306 [CIN] + 85.56 (R^2^ = 0.996) for dAdo. The CIN limit of detection (LOD) and limit of quantification (LOQ) were calculated using the equations LOD = 3.3 s/m and LOQ = 10 s/m, in which s is the standard deviation for measurements in the absence of CIN, and m is the slope of the calibration plot. Using these equations and the calibration curve, the LOD and LOQ values for CIN were found to be 0.15 and 0.48 μg mL^−1^, respectively. Moreover, the interaction was validated in terms of within-day precision of peak current of dAdo, within-day precision of peak potential of dAdo, between days precision of peak current of dAdo, and between days precision of peak potential of dAdo, in terms of RSD% values as 0.24, 0.11, 1.47, and 0.45, respectively.

The interaction of CIN with polyA was also investigated by preparing a polyA biosensor. A significant reduction in peak current of dAdo was observed after interaction of 10 µg mL^−1^ CIN on the polyA biosensor for 20 min. While the S% value was 40% in the ct-dsDNA biosensor, it was 50% in the polyA biosensor. This indicated that the polyA base was directly affected by CIN, whereas the difference between the interactions was due to the fact that the structure of ct-dsDNA was different from polyA, although, the specific base was similar.

**Figure 4:**
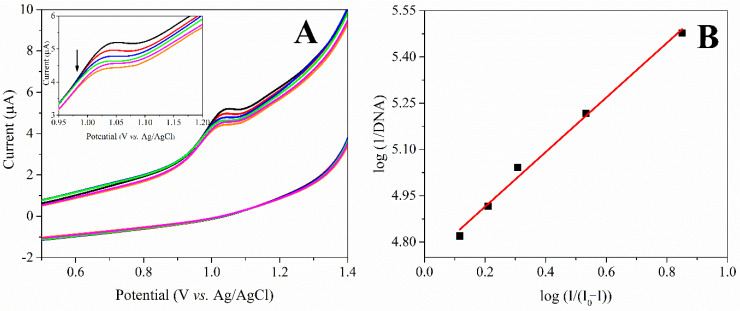
(**A**) Cyclic voltammograms of 10 μg mL^−1^ CIN and in the presence of ct-dsDNA in the range of 3.30 × 10^−6^ M–1.50 × 10^−5^ M, (**B**) Related plot of log (1/[*DNA*]) vs. *log* (*I*/(*I*_0_ − *I*)).

For a better understanding of the CIN-ct-dsDNA interaction and to determine the binding constant, an electrochemical investigation was performed in ABS (pH 4.7) using the CV method at ambient temperature [34]. When previous studies were examined, no anodic peaks were observed in the CV of ct-dsDNA in ABS (pH 4.7) [18,35]. Additionally, the CV peak current of 10 μg mL^−1^ CIN was found as 1.018 V. In order to observe the interaction mechanism of the CIN-ct-dsDNA, 10 µg mL^−1^ CIN solution was prepared in ABS (pH 4.7). Subsequently, successive ct-dsDNA concentrations in the range of 3.30 × 10^−6^ M–1.50 × 10^−5^ M were added to this solution to monitor the changes in the CV responses of CIN. It was observed that the peak current of CIN gradually decreased in the buffer solution while the increased ct-dsDNA concentrations were added to the CIN solution (Figure 4A). Based on the changes in the oxidation signal of CIN after binding to ct-dsDNA, the binding constant (K) value was determined using the following Equation (2);

(2) $$log\left(\frac{1}{\left[DNA\right]}\right)=log\;K+log\left(\frac{I}{I_{0}-I}\right)$$

where, the binding constant is expressed by *K*, and the peak current of the CIN in the absence and presence of ct-dsDNA, are represented by *I*_0_ and *I*, respectively. The *K* value is determined from the plot of *log* (1/[*DNA*]) vs. *log* (*I*/(*I*_0_ − *I*)) [36]. When the experimental results were evaluated, the binding constant of CIN and ct-dsDNA was calculated as 6.30 × 10^4^ M^−1^ (Figure 4B).

In addition, the shift in the peak potential of drug molecules in the presence of ct-dsDNA allows for determining the type of interaction between the drug and DNA. A positive shift in the potential of the drug signal indicates that the dominant mode of interaction is intercalation, while a negative potential shift indicates electrostatic interactions [11,32,37,38,39]. As seen in Figure 4A, the slight shift in peak potential may suggest that the interaction between CIN and ct-dsDNA is electrostatic or groove binding with an intercalation effect [40,41,42].

### 3.2. Interaction between CIN and ct-dsDNA Using Fluorescence Spectroscopy

**Figure 5:**
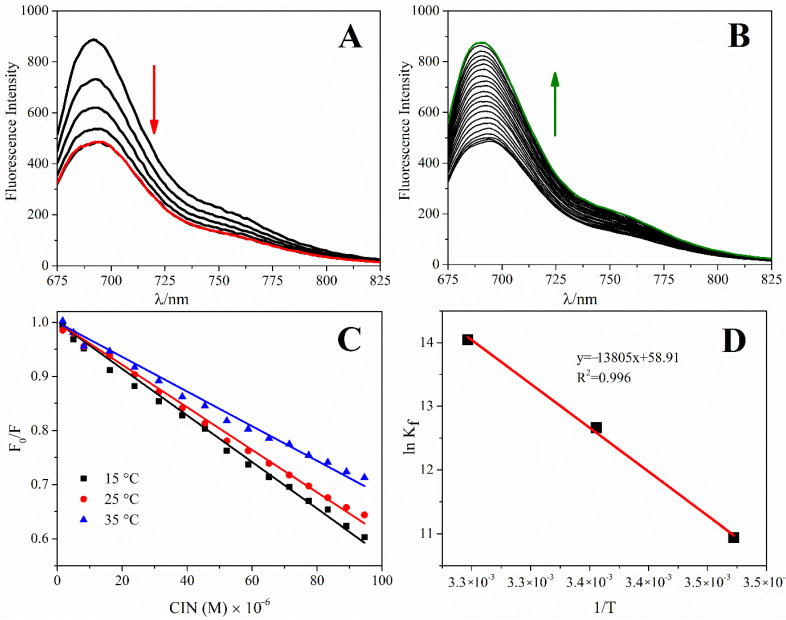
(**A**) Fluorescence spectra of MB-ct-dsDNA titration, (**B**) CIN–MB+ct-dsDNA titration in ABS (pH 4.70), λexc: 665 nm, the concentration of MB: 50 µM, the concentration of the stock solution of ct-dsDNA: 100 µg mL^−1^, (**C**) The Stern–Volmer curves at different temperatures, (**D**) Van’t Hoff plots of CIN-ct-dsDNA interactions.

Spectrofluorometric methods have been widely preferred for investigating the interaction between small organic and inorganic molecules, and dsDNA by performing fluorescence titration studies. Changes in the fluorescence signals of the molecules or dsDNA are followed to explore the interaction [43]. In the present study, the displacement approach, as a competitive approach, was used for fluorescence titrations using MB as an indicator. This approach was based on adding a molecule with higher affinity than the indicator molecule onto the dsDNA-indicator complex and replacing the indicator from the complex with the molecule by fluorescence recovery [43]. In this study, the fluorescence spectra of MB at a fixed concentration were quenched upon the addition of increasing ct-dsDNA concentrations, and it was concluded that all MB in the media bound to ct-dsDNA at the point where the signal remained constant. In the next step, increasing concentrations of CIN (3.3 × 10^−7^ M–1.9 × 10^−5^ M) were added to the ct-dsDNA-MB complex, the ct-dsDNA interacted with CIN and replaced MB, and the fluorescence signal was recovered. As shown in Figure 5A,B, a remarkable enhancement of MB + ct-dsDNA fluorescence intensity was observed with increasing concentrations of CIN. In general, the fluorescence enhancing mechanism could be categorized either as static, dynamic, or mixed quenching based on temperature. Therefore, the indicator displacement experiments were performed at three different temperatures (288 K, 298 K, and 308 K) to investigate the thermodynamic parameters. The change in fluorescence emission intensity in enhancer molecules is based on collision between molecules (dynamic) or formation of the ground-state complex (static). The Stern–Volmer constant (K_D_) determines the types of enhancing mechanisms (static, dynamic, or mixed quenching) involved in the interaction process. The fluorescence enhancement of CIN-ct-dsDNA was described by using the classical Stern–Volmer Equation (3) [44]:

(3) $$\frac{F_{0}}{F}=1-K_{D}\left[E\right]$$

where, *F*_0_ is the fluorescence emission intensity of the MB + ct-dsDNA complex at 693 nm in the absence of the CIN steady-state, and *F* is the fluorescence emission intensity of CIN-ct-dsDNA in the presence of the CIN steady-state, *K_D_* is the Stern–Volmer constant, and [*E*] is the molar concentration of CIN. The linearity of these curves clarifies the quenching type at 288 K, 298 K, 308 K. The enhancing mechanism is only static or dynamic when the curve is linear, whereas, in contrast, the enhancing is a combination of both static and dynamic types when there is a deviation from linearity. As shown in Figure 5C, the curves at different temperatures for the enhancing mechanism are linear, clearly demonstrating the single type of quenching. The increase in temperatures in static enhancing results in dissociation of the weakly bound complexes, and thus, a decrease in the enhancing constant occurs. K_D_ values at different temperatures are shown in Table 1. The above results revealed that the enhancing mechanism is static and confirms the complex formation between ct-dsDNA and CIN.

Furthermore, fluorescence titration was carried out to calculate the binding constant (K_f_) and binding sites (n) for the interaction between CIN and ct-dsDNA. The change of fluorescence emission intensity at 693 nm was used to calculate K_f_ and n for the binding of CIN to ct-dsDNA from the following Equation (4):

(4) $$log\frac{\left(F-F_{0}\right)}{F}=\log K_{f}+n\;log\;\left[E\right]$$

The values of *K_f_* and *n* for the [CIN-ct-dsDNA] complex are given in Table 1. According to the results, approximately one binding site on ct-dsDNA for CIN was found with high binding stability. Furthermore, the binding constants for the CIN-ct-dsDNA interaction were much higher than those observed for the MB-ct-dsDNA interaction (at the level of ~10^4^).

Finally, to calculate thermodynamic parameters, binding constants computed at 288 K, 298 K, and 308 K were used to construct a Van’t Hoff plot. The Van’t Hoff plot of ln *K_f_* vs. 1/T (Figure 5C,D) was employed to determine Δ*H* and Δ*S* (Equation (5)). According to the binding constants at 288 K, 298 K, and 308 K, the free energy change (Δ*G*) was calculated using Equation (6).

(5) $$\ln K=-\frac{\Delta H}{RT}+\frac{\Delta S}{R}$$

(6) ΔG=ΔH−TΔS

where *K* is the binding constant as *K_f_* at the related temperature and *R* is the gas constant.

Hydrophobic forces, electrostatic interactions, hydrogen bonds, or van der Waals interactions can be significant interaction forces between pharmaceuticals and biomolecules. The type of interaction between these molecules and biomolecules can be clarified by evaluating the values of enthalpy change (Δ*H*) and entropy change (Δ*S*) [43]:

(i) for hydrophobic forces, Δ*H*  >  0 and Δ*S*  >  0;
(ii) for van der Waals interactions and hydrogen bonds, Δ*H*  <  0 and Δ*S*  <  0;
(iii) for electrostatic interactions, Δ*H*  <  0 and Δ*S*  >  0.

The positive values of Δ*H* and Δ*S* explained that hydrophobic forces play a primary role in the binding of CIN to ct-dsDNA. The negative Δ*G* value manifested that the interaction process between CIN and ct-dsDNA is spontaneous at all temperatures (Table 1).

### 3.3. Molecular Docking Studies for the Interaction of CIN and ct-dsDNA

**Figure 6:**
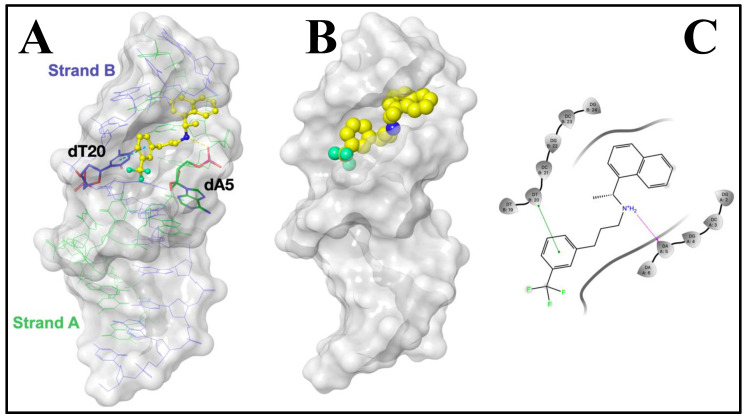
The proposed binding mode of CIN represented as: (**A**) Yellow ball and stick mode; (**B**) CPK mode with dsDNA (represented as gray surface) (PDB: 1BNA) illustrating the interactions observed. Hydrogen bond and π-π contact are represented as: (**A**) Yellow and cyan dotted lines, respectively; (**C**) (in 2D-interaction map) Magenta and green lines, respectively.

CIN was docked into the dsDNA structure in order to predict the preferred orientation of the CIN inside the dsDNA based on binding energy using the Glide module implemented in the Schrödinger Small-Molecule Drug Discovery Suite. According to the results, CIN interacted with dsDNA by (i) H-bonding formed (2.04 Å) between its amine group and phosphate carbonyl of deoxyadenosine base (dA5) in the strand A; and (ii) π-π stacking contact formed (4.91 Å) between its phenyl ring and pyrimidine ring of deoxythymidine base (dT20) in the strand B. The minimized free energy of the CIN-dsDNA docked structure was found to be −6.14 kcal mol^−1^. The binding conformation and minor groove interactions of CIN are given in Figure 6.

## 4. Conclusions

Investigation of interactions with DNA plays an important role in the design of new drugs. Analytical techniques are frequently used in drug–DNA interaction studies to reduce the side effects of drugs, as well as to develop targeted and efficient drugs.

The drug–DNA interaction can be interpreted by examining the changes in the electroactive bases of DNA before and after the interaction of the drug with electrochemical DNA biosensors. In this study, numerous advantages such as practicality, low cost, and sensitivity offered by electrochemical techniques and DNA biosensors were used to examine drug–DNA interactions. An interaction study of DNA with SPTH drug CIN was carried out by constructing a ct-dsDNA biosensor on the GCE surface, and the decrease in the dAdo peak current of ct-dsDNA was observed at 1.22 V after the interaction. A similar study was carried out with polyA, which could only observe the dAdo oxidation peak, and the interaction with CIN was determined electrochemically. Electrochemical signal reductions resulting from the interaction of ct-dsDNA and polyA with CIN were compared. It was confirmed that CIN binds to dADo and the binding mode was found to be groove binding with an intercalation effect to ct-dsDNA.

In addition, the interaction of MB-ct-dsDNA complex with CIN was investigated by the widely used spectrofluorimetric method. The significant change in fluorescence intensity of the MB-ct-dsDNA complex when exposed to CIN and the calculated binding constant value (at the level of ~10^4^) provided substantial evidence that the drug interacts with DNA. Thermodynamic data was calculated according to the results of the fluorescence study performed at different temperatures, and it was concluded that the strengthening mechanism originating from the complex structure formed by CIN-MB-ct-dsDNA was realized statically. According to the calculated Δ*H* and Δ*S* values, it can be said that hydrophobic forces were involved in the binding of CIN and ct-ds-DNA. In addition, the calculated negative Δ*G* values may explain the interaction between CIN and ct-dsDNA spontaneously and the determination of the interaction type being the groove-binding mode. Molecular docking simulation studies, which are mostly used in drug design and provide important information about drug–DNA interaction, show that the binding mode is found as a result of the H bond formed between the phosphate carbonyl on the bases of deoxyadenosine and the amine group of CIN, and the π-π stacking contact between CIN and dsDNA.

## Figures and Tables

**Table 1:** Stern–Volmer constants, binding constants, binding sites, and thermodynamic parameters.

Temperature (K)	Enhancement Constants Value		Binding Constants Value				ln K_f_ (M^−1^)	Δ*G*° (kJ mol^–1^)	Δ*H*° (kJ mol^–1^)	Δ*S*° (J mol^–1^ K^–1^)
	R^2^	K_D_ (M^−1^)	R^2^	n	K_f_	log K_f_				
288	0.999	21,541.0	0.998	1.11	5.62 × 10^4^	4.75	10.94	−26.28	114.78	489.78
298	0.999	19,669.0	0.993	1.24	3.16 × 10^5^	5.50	12.66	−31.17		
308	0.999	16,017.0	0.992	1.39	1.26 × 10^6^	6.10	14.05	−36.07		

## Data Availability

Not applicable.
